# Letter in response to Colpani et al “A comparative study in type 2 von Willebrand disease patients using four different platelet-dependent von Willebrand factor assays”

**DOI:** 10.1016/j.rpth.2023.100287

**Published:** 2023-06-28

**Authors:** Emmanuel J. Favaloro

**Affiliations:** 1 Department of Haematology, Sydney Centres for Thrombosis and Haemostasis, Institute of Clinical Pathology and Medical Research, NSW Health Pathology, Westmead Hospital, Westmead, New South Wales, Australia; 2 School of Dentistry and Medical Sciences, Faculty of Science and Health, Charles Sturt University, Wagga Wagga, New South Wales, Australia; 3 School of Medical Sciences, Faculty of Medicine and Health, University of Sydney, Westmead Hospital, Westmead, New South Wales, Australia

To the Editor,

I was interested to read the recent original study by Colpani et al. [1], who undertook a comparative study in patients with type 2 von Willebrand disease (VWD) using 4 different platelet-dependent von Willebrand factor (VWF) assays. I do not intend to criticize the study, which was an interesting analysis of result patterns using VWF activity assays, essentially reflecting platelet glycoprotein Ib (GPIb) binding (“VWF:GPIbB”) although I was surprised that some key VWF activity assays were omitted from the study. The authors assessed VWF ristocetin cofactor (VWF:RCo) by aggregometry and by an automated method and the gain of function VWF assay using mutated GPIb (VWF:GPIbM) by ELISA and an automated method as well as VWF antigen (VWF:Ag). Collagen binding (VWF:CB) and any VWF:GPIbB assay using recombinant GPIb (VWF:GPIbR) were not performed. This study was performed in part to assess the utility of VWF activity/Ag ratios to capture type 2 VWD cases, particularly relevant in the age of the latest diagnostic VWD guidelines [2], which recommend use of newer VWF:GPIbB assays (VWF:GPIbM or VWF:GPIbR) ahead of classical VWF:RCo since they would likely yield lower variability, better low VWF level sensitivity, and improve VWD diagnosis reliability. The guidelines also recommend a VWF activity/Ag ratio cut-off of 0.7, ahead of 0.5, to avoid missing type 2 VWD [2]. Although agreeing 0.7 is a better cut-off than 0.5, recent data highlights that optimal cut-offs are assay dependent, and using chemiluminescence assays, for example, 0.6 provides the best cut-off value with total separation of type 1 vs 2 VWD [3]. Like us, Colpani et al. [1] classically used a cutoff of 0.6 in their laboratory [1], but yielding potential misidentifications of type 2B VWD, they stated that a VWF activity/Ag ratio of 0.7 halved the number of “misdiagnosed” patients. Although true, this only tells half the story since the authors did not include any type 1 VWD cases in the data set. Thus, capturing more type 2B VWD with a cut-off of 0.7 (improved sensitivity) also risks falsely capturing more type 1 VWD cases as type 2 (ie, reduced specificity). Cut-off values ultimately represent trade-offs between capturing true positives (ie, type 2 VWD) vs capturing false positives (ie, type 1 VWD). If the target is to capture more type 2 VWD cases, as per the guidelines [2], then 0.7 will achieve this but it will also falsely capture more type 1 cases. Indeed, for the automated VWF:GPIbM assay, a cut-off of 1.1 would have been required to capture all the type 2B VWD cases (see Table 1 in [1]), which interestingly would have also captured all the healthy subjects. In conclusion, Colpani et al. [1] did not identify which cut-off value (0.7 or 0.6) best discriminated between type 1 and 2 VWD, and their finding that fewer type 2 cases were missed using a cut-off of 0.7 is entirely expected. What is missing is the “cost” of this capture of type 2 VWD in terms of false type 1 VWD capture, as highlighted in Figure.

**Figure:**
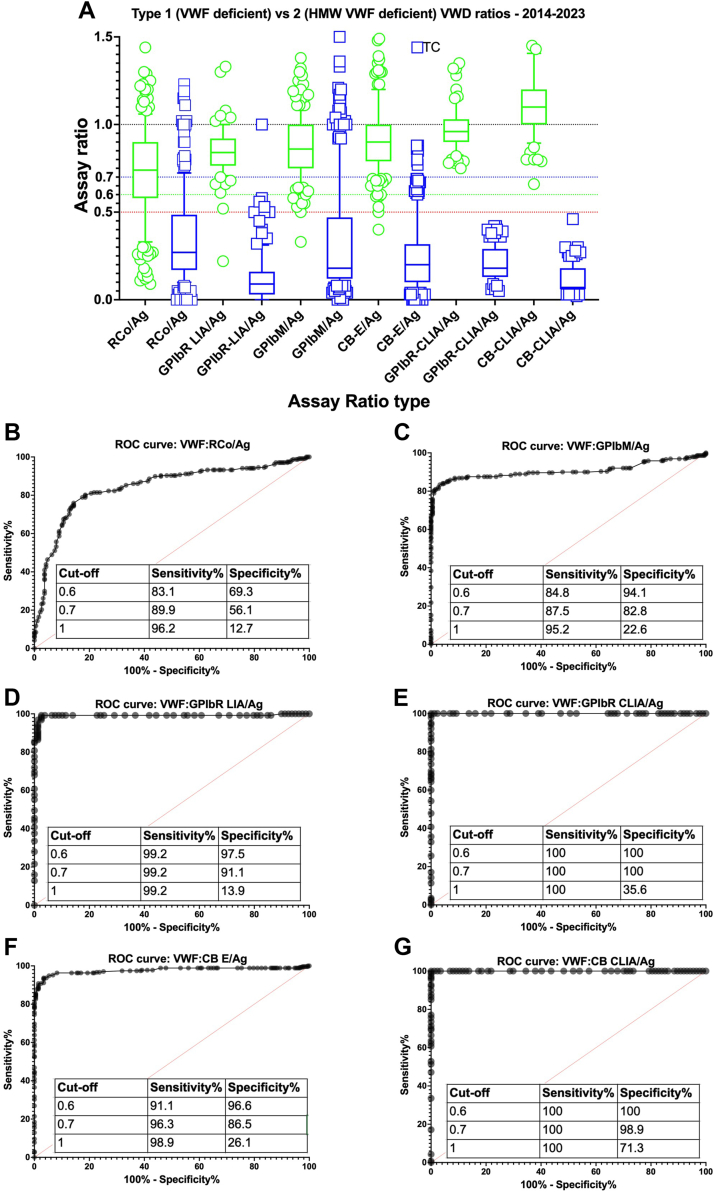
(A) Composite of contemporary data for von Willebrand factor (VWF) activity/antigen (Ag) assay ratios as reported by participants of the Royal College of Pathologists of Australasia Quality Assurance Program. Ratios reported by laboratories for VWD cases ascribed to type 1 (“VWF deficient”; green) vs type 2 (“high molecular weight VWF deficient”; blue) for all samples assessed between 2014 and 2023 (to date). Data are shown as boxes, and whiskers show median and 10th to 90th percentiles, with outliers shown by symbols. (B–G) Separate receiver operator curves (ROCs) for each ratio type. Chemiluminescence (CLIA)-based assay ratios (VWF:GPIbR/Ag and VWF:CB/Ag) showed complete separation of data, with 0.6 identified as the optimal cut-off value. Reasonable separation of group data was also seen with VWF:GPIbR/Ag (latex agglutination; LIA) and collagen binding (VWF:CB)/Ag (ELISA; E) ratios. In contrast, more overlap in ratios was observed using VWF:RCo/Ag and VWF:GPIbM/Ag ratios. Here, cut-offs of 0.7 captured more type 2 VWD cases but also more type 1 VWD cases. Tables within each figure show relative sensitivity vs specificity values for cut-offs of 0.6, 0.7, and 1.0. CB, collagen binding; GPIbM, recombinant mutated platelet glycoprotein Ib; GPIbR, recombinant platelet glycoprotein Ib; HMW, high molecular weight; RCo, ristocetin cofactor; VWD, von Willebrand disease.
